# Cerebrospinal fluid and blood flow in mild cognitive impairment and Alzheimer's disease: a differential diagnosis from idiopathic normal pressure hydrocephalus

**DOI:** 10.1186/2045-8118-8-12

**Published:** 2011-02-17

**Authors:** Soraya El Sankari, Catherine Gondry-Jouet, Anthony Fichten, Olivier Godefroy, Jean Marie Serot, Hervé Deramond, Marc Etienne Meyer, Olivier Balédent

**Affiliations:** 1 Department of Image Processing, Jules Verne University of Picardy and Amiens University Hospital, CHU d'Amiens, F-80054 Amiens cedex, France; 2 TIDAM research unit, Jules Verne University of Picardy and Amiens University Hospital, CHU d'Amiens, F-80054 Amiens cedex, France; 3 Department of Neurology, Jules Verne University of Picardy and Amiens University Hospital, CHU d'Amiens, F-80054 Amiens cedex, France; 4 Department of Radiology, Jules Verne University of Picardy and Amiens University Hospital, CHU d'Amiens, F-80054 Amiens cedex, France; 5 Department of Neurosurgery, Jules Verne University of Picardy and Amiens University Hospital, CHU d'Amiens, F-80054 Amiens cedex, France; 6 Department of Geriatrics Jules Verne University of Picardy and Amiens University Hospital, CHU d'Amiens, F-80054 Amiens cedex, France

## Abstract

**Background:**

Phase-contrast magnetic resonance imaging (PC-MRI) enables quantification of cerebrospinal fluid (CSF) flow and total cerebral blood (tCBF) flow and may be of value for the etiological diagnosis of neurodegenerative diseases. This investigation aimed to study CSF flow and intracerebral vascular flow in patients with Alzheimer's disease (AD) and patients with amnesic mild cognitive impairment (a-MCI) and to compare the results with patients with idiopathic normal pressure hydrocephalus (NPH) and with healthy elderly volunteers (HEV).

**Methods:**

Ten a-MCI and 9 mild AD patients were identified in a comprehensive neurological and neuropsychological assessment. They underwent brain MRI; PC-MRI pulse sequence was performed with the following parameters: two views per segment; flip angle: 25° for vascular flow and 20° for CSF flow; field-of-view (FOV): 14 × 14 mm²; matrix: 256 × 128; slice thickness: 5 mm; with one excitation for exams on the 3 T machine, and 2 excitations for the 1.5 T machine exams. Velocity (encoding) sensitization was set to 80 cm/s for the vessels at the cervical level, 10 or 20 cm/s for the aqueduct and 5 cm/s for the cervical subarachnoid space (SAS). Dynamic flow images were analyzed with in-house processing software. The patients' results were compared with those obtained for HEVs (n = 12), and for NPH patients (n = 13), using multivariate analysis.

**Results:**

Arterial tCBF and the calculated pulsatility index were significantly greater in a-MCI patients than in HEVs. In contrast, vascular parameters were lower in NPH patients. Cervical CSF flow analysis yielded similar values for all four populations. Aqueductal CSF stroke volumes (in μl per cardiac cycle) were similar in HEVs (34 ± 17) and AD patients (39 ± 18). In contrast, the aqueductal CSF was hyperdynamic in a-MCI patients (73 ± 33) and even more so in NPH patients (167 ± 89).

**Conclusion:**

Our preliminary data show that a-MCI patients present with high systolic arterial peak flows, which are associated with higher mean total cerebral arterial flows. Aqueductal CSF oscillations are within normal range in AD and higher than normal in NPH. This study provides an original dynamic vision of cerebral neurodegenerative diseases, consistent with the vascular theory for AD, and supporting primary flow disturbances different from those observed in NPH.

## Introduction

Although the exact frequencies of different types of dementia are still difficult to assess, Alzheimer's disease (AD) is the most common subtype and idiopathic normal pressure hydrocephalus (NPH) is rare [1]. Subtle losses of cognitive function (particularly memory complaints, contrasting with preserved daily living activities), can result from normal ageing but may also represent a transitional state to early AD and are frequently referred to as amnesic mild cognitive impairment (a-MCI) [2]. Although the reported prevalence of a-MCI varies from one study to another partly as a result of different evaluation criteria, this condition is now recognized as a risk factor for AD. The annual conversion rate from a-MCI to AD is estimated between 10 and 15%, compared with 1 to 2% seen in healthy, non -impaired control subjects [2].

Clinicians routinely use morphological brain imaging especially magnetic resonance imaging (MRI), to study etiologies and assist with differential diagnosis in patients with cognitive disorders. These conventional techniques have revealed various degrees of ventricular dilation, with global or localized cerebral atrophy. In some patients, cerebral and/or ventricular dilation may be difficult to relate to any of the various etiologies considered. Even though the cortical atrophy observed in AD patients occurs in a defined sequence which is increasingly well understood [3], it remains the case that volume loss detected on MRI is related to the extent of neuron loss [4]. Thus, it may be difficult to distinguish age-related atrophy from atrophy caused by mild AD.

In normal ageing, the overall volume of the brain compartment falls slightly. However, this atrophy is significantly greater in patients with AD - by up to 25 or 50%, depending on the severity of the disease. The atrophy predominates in the temporal regions and especially in the hippocampus [5,6]. Recent publications have suggested similar local atrophy in MCI patients [6] and longitudinal studies have emphasized the potential value of rapid temporal volume changes in predicting conversion from MCI to AD [4,7]. In contrast, NPH is typically associated with ventricular dilation far in excess of that expected from cortical atrophy.

Several techniques have been used to study the effects of normal ageing on vascular flow. Low global cerebral blood flow in the elderly has been variously reported by studies using sonography [8], positron emission tomography (PET) [9], angiographic MRI [10,11] and, more recently, phase contrast (PC)-MRI [12,13]. Metabolic studies suggest the presence of vascular modifications in AD patients and in a-MCI patients who will convert to AD [13,14]. Similarly, a role for vascular flow in the pathogenesis of NPH has been suggested in the last decade, initially with an emphasis on the involvement of ischemia of the deep white matter [15]. The major role of venous circulation in the regulation of intracerebral dynamics has been defended by other authors [16] and has recently led to the "venous hypothesis of NPH" [17], in which venous dysfunction is presumed to be a crucial initiating factor for hydrocephalus.

New theories have suggested that senescence of the CSF circulatory system may contribute to the pathogenesis of neurodegenerative diseases [18] and that there may be a metabolic and neuropathological continuum which parallels the observed clinical overlap. Morphological studies have shown structural alterations of the choroid plexuses in AD patients, with reduced CSF secretion of proteins [19]. Although it has been suggested that these structural and metabolic modifications induce dynamic alterations in the intracranial fluids, the few studies that have investigated (at least in part) these features have generally featured small populations [20,21]. Some authors further suggest that AD is related to a dramatic decrease in CSF production, which slows beta amyloid (Aβ) clearance, increases Aβ deposition in the meninges and thus raises the resistance to CSF outflow [22,23]. In a randomized, controlled pilot study [24], the same authors suggested that there is a subgroup of "hybrid" AD-NPH patients who meet the clinical criteria for AD and also have elevated CSF pressure suggesting early-stage hydrocephalus. Thus, the clinical overlap between these etiologies and the presence of AD-like neuropathological features in NPH patients varying from 27 to 75%, [25,26] depending on the severity of dementia, suggest a common physiological basis for CSF circulatory failure - an effect which is far greater than the decrease in turnover observed during normal ageing.

A recent study [12] in healthy elderly volunteers (HEVs) showed lower pulsatility in CSF oscillations in both ventricular and subarachnoid compartments, when compared to healthy young volunteers; this contrasts with the hyperdynamic aqueductal CSF oscillations widely described in NPH patients [21,27,28]. In the present study, we set out to compare CSF flow and cerebral blood flow in a-MCI and AD patients, compared to NPH patients and HEVs, with the aim of developing tools for use in differential diagnosis.

## Materials and methods

### Participants

Patients were recruited from our hospital's Alzheimer's disease patient registry and research centre. At baseline, all patients met the criteria for a-MCI or AD. Patients were categorized in a special consultation with a neurologist and a neuropsychologist. Dementia and AD were diagnosed according to the DSM-IV and NINCDS-ADRDA criteria [29], respectively. We selected patients with mild AD, corresponding to a Mini Mental State Examination (MMSE) [30] score ≥ 20 out of 30. The diagnostic criteria for elderly patients with a-MCI were those published by Petersen *et al*. [2]: (i) a subjective memory complaint (i.e. reported by the patient or by his family), (ii) a documented, objective memory impairment adjusted for age and education, (iii) relatively normal general cognitive function, (iv) no dementia according to the DSM-IV criteria, and (v) generally preserved activities of daily living. Patients with a relevant neurological disease (stroke, tumour, etc.) or cerebrovascular risk factors (except for arterial hypertension controlled by medication) were not included in the study.

The third (NPH) group consisted of age-matched patients presenting with 2 or 3 of the Hakim triad symptoms (gait troubles were mandatory) with an insidious onset, no antecedent events such as head trauma, intracerebral haemorrhage or meningitis or other known cause of secondary hydrocephalus, ventricular enlargement on brain MRI (Evan's ratio > 0.3) and no macroscopic signs of obstruction to CSF flow. Based on these criteria and published guidelines [31], patients were classified as having "possible" NPH and the diagnosis was confirmed by specialist neurosurgical consultation and positive tap tests.

Hence, our patient populations consisted of 10 a-MCI patients (mean ± standard deviation (SD) age: 78 ± 7 years), 9 AD patients (mean ± SD age: 79 ± 5 years) and 13 NPH patients (mean ± SD age: 70 ± 6 years). The groups' demographic data and MMSE scores are summarized and compared in Table 1. All patients underwent the same PC-MRI imaging protocol. The patients were compared with a population (n = 12) of age-matched HEVs (mean ± SD age: 71 ± 9 years) with normal neurological and neuropsychological screening results (notably a normal MMSE score for educational level) and no active psychiatric, neurological or cognitive disorders.

**Table 1:** Demographic data and results for vascular and CSF flows in four groups

Characteristic	HEVs	aMCI	AD	NPH	*P*-value
Number of subjects	12	10	9	13	*NS*

Age *(years*)	71 ± 9	78 ± 7	79 ± 5	70 ± 6	*NS*

Disease duration (months)	/	27 ± 18	29 ± 11.4	24 ± 15	*NS*

MMSE	29 ± 2	26 ± 3	21 ± 3	ND	<0.05

BPM	78 ± 10	65 ± 8	72 ± 8	73 ± 15	*NS*

tCBF (*ml/min*)	509 ± 108	638 ± 126	560 ± 83	450 ± 129	<0.01

API	59 ± 12	119 ± 69	107 ± 50	93 ± 42	<0.05

Arterial pulse volume *(ml)*	0.8 ± 0.3	1.4 ± 0.4	1 ± 0.3	0.8 ± 0.4	<0.01

VBF (*ml/min*)	367 ± 130	522 ± 157	457 ± 71	361 ± 92	0.01

AV stroke volume (*ml/CC*)	0.7 ± 0.3	1.3 ± 0.3	1 ± 0.5	0.9 ± 0.4	0.01

AV delay (% of CC)	18 ± 6	12 ± 6	8 ± 7	10 ± 4	<0.01

Cervical stroke volume (*μl/CC*)	457 ± 154	584 ± 152	450 ± 221	455 ± 133	*NS*

Aqueductal stroke volume (*μl/CC*)	34 ± 17	73 ± 33	39 ± 18	167 ± 89	<0.01

Results are given for four groups: (i) healthy elderly volunteers (HEVs) and patients with (ii) amnesic mild cognitive impairment (a-MCI), (iii) Alzheimer's disease (AD) and (iv) normal pressure hydrocephalus (NPH). Values are expressed as the mean ± standard deviation. MMSE: Mini Mental State Examination; BPM: heart rate in beats per minute; tCBF: total cerebral arterial blood flow; API: arterial pulsatility index; VBF: venous blood flow; AV: arteriovenous; ml: millilitre; μl: microlitre; min: minute; CC: cardiac cycle.

The threshold for statistical significance was set to *p *< 0.05; NS: not significant.

The study protocol was approved by the local independent ethics committee. All participants had received an explanation of the study's objectives and procedures and provided their written informed consent to participation.

### Data acquisition

**Figure 1:**
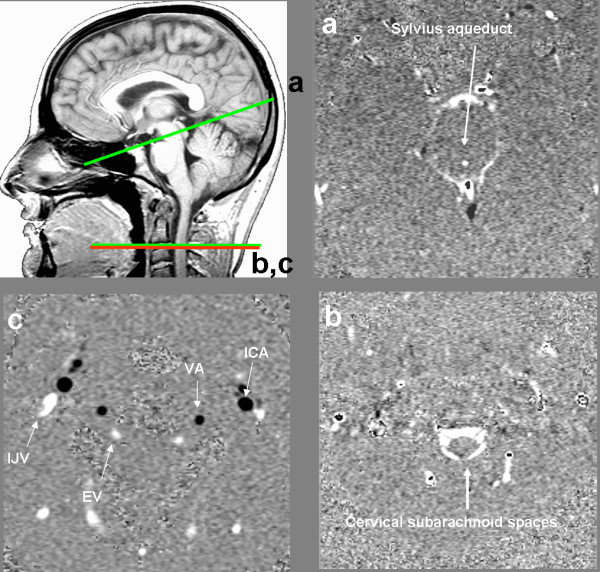
**Data acquisition by phase-contrast magnetic resonance imaging**. Sagittal scout view sequences were used as localizers to select the anatomical levels for flow quantification. The selected acquisition planes were perpendicular to the presumed flow direction. Sections through the aqueduct of Sylvius (a) and the C2-C3 subarachnoid space (b) were used for CSF flow measurement. By varying the velocity encoding, the same cervical section level (c) was used to measure vascular flows in the left and right internal carotid arteries (ICAs), vertebral arteries (VAs), internal jugular veins (IJVs) and epidural veins (EVs). Figures 1(a), (b) and (c) represent the flow acquisition over the cardiac cycle. See also additional file: Movie1 for the original data used to perform this analysis'.

All MRI exams were performed with a standardized imaging protocol on a 1.5 or 3 Tesla (T) machine (Signa; General Electric Medical System, Milwaukee, WI, USA). Flow images were acquired with a 2 D fast cine PC-MRI pulse sequence with retrospective peripheral gating, so that the 32 analyzed frames covered the entire cardiac cycle (CC). The MRI parameters were as follows: 2 views per segment; flip angle: 25° for vascular flow and 20° for CSF flow; field-of-view (FOV): 14 × 14 mm²; matrix: 256 × 128; slice thickness: 5 mm; one excitation for the 3 T machine and two for the 1.5 T machine. Velocity (encoding) sensitization was set to 80 cm/s for the vessels, 10 or 20 cm/s for the aqueduct and 5 cm/s for the cervical subarachnoid space (SAS). A sagittal scout view was used as a localizer to select the anatomical levels for flow quantification. The selected acquisition planes were perpendicular to the presumed flow direction. Sections for each flow series are presented in Figure 1. The acquisition time for each flow series was about 90 s on the 3 T machine and 180 s on the 1.5 T machine with slight variations that depended on the participant's heart rate. The total, additional examination time for these CSF and blood flow investigations was around 10 min.

### Data analysis

**Figure 2:**
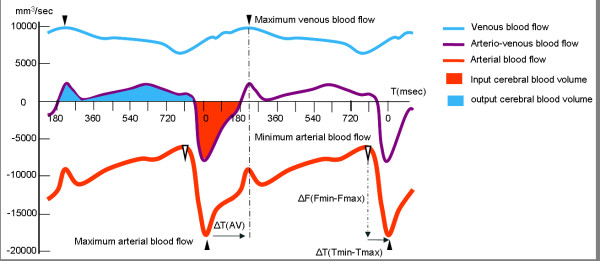
**Vascular arterial, venous and arteriovenous flow curves **. Mean arterial, venous and arteriovenous flows are represented over two successive cardiac cycles (CC) in one patient. The arterial blood flow peaks and troughs are represented. The difference in amplitude (ΔF = F_max _- F_min_) and latency ((ΔT = T_min _- T_max_) between these 2 features is also shown. The arterial pulsatility index (defined as ΔF/ΔT) corresponds to the slope of the arterial flow curve at the beginning of systole. The arterial pulse volume (defined as ΔF × ΔT/2) corresponds to the systolic arterial inflow volume. The arteriovenous flow curve results from the difference between the arterial and the venous flow curves over a given CC. Integration of the area under the curve yields the arteriovenous stroke volume, which represents the volume blood carried into the cranium (the input cerebral blood volume) or expulsed caudally (the output cerebral blood volume) from the cranium over the course of the CC. The difference in latency between the arterial and venous flow peaks (in milliseconds or as a percentage of the CC) corresponds to the arteriovenous delay (AVD).

Data were analyzed using in-house image processing software http://www.tidam.fr. An optimized CSF and blood flow segmentation algorithm was used to automatically extract the region of interest (ROI) at each level. The ROIs (Figure 1) considered here were the right and left internal carotid arteries (ICAs), vertebral arteries (VAs), internal jugular veins (IJVs) and epidural veins (EVs) (for blood flows) and the aqueductal and cervical (C2-C3) areas (for CSF flows). In each ROI, flows were calculated for each of the 32 time frames in order to build a flow curve over the course of the CC. Flows in the ICA and VA on the left and right sides were summed to generate the total arterial cerebral blood flow (tCBF). We also calculated an arterial pulsatility index (API), which corresponded to the slope of the flow curve over time. We first measured the maximum and minimum flow amplitudes (F_max _and F_min_, respectively, expressed in ml/min) and their times of occurrence (T_max _and T_min_, respectively, expressed in milliseconds). The API index was then calculated as follows: API = (F_max _- F_min_)/(T_min _- T_max_)/2, and is represented in Figure 2.

We also used these parameters (F_max _- F_min _and T_min _- T_max_) to calculate the arterial volume pulsed at the beginning of systole, which is expressed as follows (in ml):

Arterial pulse volume = (F_max _- F_min_) × (T_min _- T_max_). Similarly, the cervical venous blood flow (VBF) was calculated by summing the venous flows in the left and right IJVs and EVs. A correction factor was applied to the amplitude of the internal jugular flow so that the mean total inflow arterial rate equalled the mean total outflow venous rate. The difference in total arterial and total venous flows over a CC corresponds to the arteriovenous (AV) curve, which is shown in Figure 2. After integration, the area under the curve represents the AV stroke volume (SV) and corresponds to the total blood volume mobilized in either caudal (output) or cranial (input) directions during the CC. In addition, we calculated the cervical arteriovenous delay (AVD), which corresponds to the time difference between the arterial systolic maximum flow peak (in the ICA) and the venous maximum peak (in the IJV). The AVD is expressed in milliseconds or as a percentage of the CC. Lastly, analysis of the CSF flow curves at aqueductal and cervical levels yielded the CSF SVs, which represent the oscillatory CSF volume displaced in both directions through the given ROI at each level [32].

An additional file presents cerebral flows animation (Additional file 1. 3-D global animation of CSF and cerebral vascular flows). This video of an NPH patient was created by merging PC-MRI data sets with morphological MR images. It shows animated CSF/blood flows through the aqueduct of Sylvius, the C2-C3 subarachnoid spaces, the internal and vertebral carotid arteries and the jugular veins during the cardiac cycle.

### Statistical analysis

Comparison of CSF and blood flow parameters in the four groups (AD, a-MCI and NPH patients and HEVs) was based on standard analysis of variance (ANOVA) for parametric data (tCBF, VBF, AVD, AV SV and cervical SV) and a Kruskal-Wallis one-way ANOVA on ranks for non-parametric data (API and aqueductal SV). When the ANOVA result achieved statistical significance, Bonferroni *post-hoc *analysis was used to identify the significantly different groups. The threshold for statistical significance was set to 0.05.

## Results

Flow results for the arterial, venous and CSF compartments in the four groups are summarized in Table 1 and details of *post hoc *pair-wise comparisons are shown in Table 2.

**Table 2:** Post hoc comparisons of vascular and CSF flow results in the four groups.

	HEVs vs. aMCI	HEVs vs. AD	HEVs vs. NPH	aMCI vs. AD	aMCI vs. NPH	AD vs. NPH
tCBF	**0.01**	NS	NS	NS	**<0.01**	0.06

API	**<0.05**	NS	NS	NS	NS	NS

Arterial pulse volume	**<0.01**	NS	NS	0.06	**<0.01**	NS

VBF	**<0.01**	NS	NS	NS	**<0.01**	NS

AV stroke volume	**<0.01**	NS	NS	NS	0.05	NS

AV delay	**<0.01**	**<0.01**	**<0.01**	NS	NS	NS

Aqueductal stroke volume	NS	NS	**<0.05**	NS	NS	**<0.05**

HEVs: Healthy elderly volunteers; aMCI: patients with amnesic mild cognitive impairment; AD: patients with Alzheimer's disease; NPH: patients with normal pressure hydrocephalus; tCBF: total cerebral arterial blood flow; API: arterial pulsatility index; VBF: venous blood flow; AV: arteriovenous. *P *values in bold are statistically significant. *P *values on a grey background are close to statistical significance. NS: not significant.

**Figure 3:**
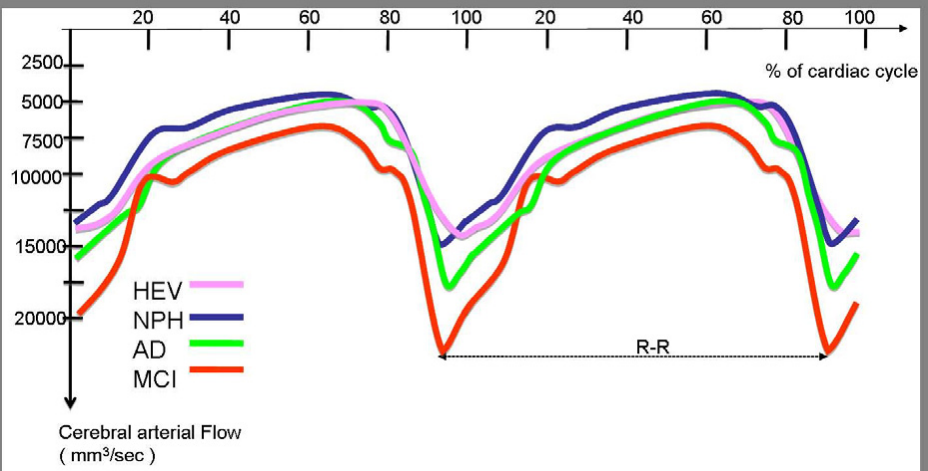
**Arterial flow curves in the three groups of patients and in healthy volunteers**. The mean arterial flow curve is plotted for each of the four populations over two successive cardiac cycles. In order to simplify the figure, the standard deviations have been intentionally omitted. The flow scale increases from top to bottom. Note the sharper, higher systolic arterial peak in MCI and (to a lesser extent) in AD patients, when compared with healthy elderly volunteers (HEVs) and NPH patients. There were significant differences between these curves (please refer to the values reported in Tables 1 and 2).

The mean arterial (tCBF) flow curves for each of the four populations are shown in Figure 3.

Vascular flow in a-MCI patients showed significant differences when compared with HEVs, with greater mean arterial and venous flow and a greater AV SV. Similarly, we observed hyperdynamic arterial flow as represented by the sharp peak seen in Figure 3. This finding was confirmed by the significantly elevated API and arterial pulse volume found in a-MCI patients. Although the aqueductal CSF SV was much greater in a-MCI patients than in HEVs, the difference did not achieve statistical significance. This was also the case for the cervical CSF SV, despite higher values than in the other three groups.

In AD patients, vascular flow was greater than in HEVs and NPH patients but was less dynamic than in a-MCI patients. The latter difference did not achieve statistical significance (Table 2). The ventricular CSF SV was similar to that seen in HEVs; hence, AD patients did not show the elevated aqueductal CSF oscillation observed in a-MCI patients. In contrast, NPH patients showed lower mean arterial and venous flows. These values did not differ significantly from those recorded in HEVs but were significantly lower than those observed in a-MCI patients. The ventricular CSF SV was higher in NPH patients than in all of the other groups and this difference achieved statistical significance when comparing NPH patients with HEVs and to AD patients.

Although both a-MCI and NPH patients showed a lower than normal AVD, the two groups could be differentiated on the basis of vascular data: a-MCI patients had hyperdynamic arterial and venous flows, whereas NPH patients had lower mean arterial and venous flows and less arterial pulsatility. Finally, the AVD was significantly lower in all patient groups than in HEVs.

## Discussion

**Figure 4:**
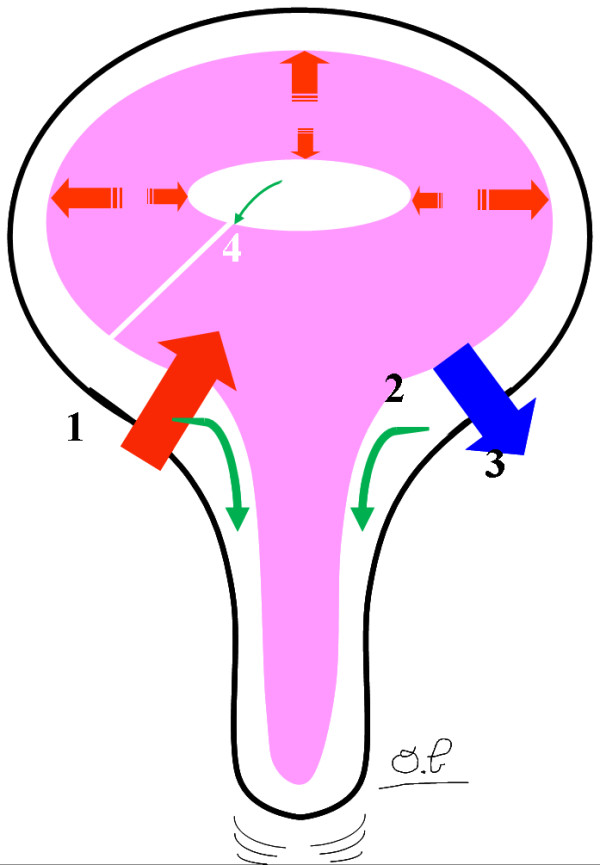
**The dynamic interaction between the intracranial compartments**. At the start of systole, an arterial volume suddenly flows into the cranium. This causes an immediate increase in the intracranial pressure (ICP). According to the Monro-Kellie doctrine, this increase in ICP is countered by a succession of flush flows through the venous and CSF compartments. The temporal coordination of these flush flows is now well documented and is organized according to the venous and CSF viscosities and flow resistances and brain compliance. The arterial peak flow (1) is first transmitted to the cervical CSF flow (2), the venous blood flow (3) and, lastly, the ventricular CSF flow (4).

Several authors [27,33-35] have suggested that cerebral atrophy or hydrocephalus can be induced by an imbalance in the interaction between three intracranial compartments: the brain parenchyma, the ventricular and subarachnoid CSF and the vascular tree (which extends from the arteries to the venous system and includes the capillary vasculature) (Figure 4). These three compartments are supposedly incompressible in steady physiological states and the adult cranium is usually considered as a rigid, closed box. As such, these systems are governed by the Monro-Kellie doctrine, which states that any volume increase in one compartment should be compensated by the withdrawal of an equal volume in one of the other two compartments, in order to maintain a steady intracranial pressure (ICP) [34,36,37]. Hence, each of these compartments can be characterized by its compliance - an indicator of distensibility, which corresponds to the pressure increase that occurs in the system when a volume is added to it.

To a certain extent, this pressure equilibrium is threatened at the beginning of each systole, when a high volume of blood is carried into the cranium (corresponding to the arterial pulse volume). A rapidly movable volume should therefore exit the cranium; this represents the "total cranial mobile compliance", the role of which is to avoid irreversible brain damage [36,37]. Thus, transmission of the pressure wave from the arterial tree to the venous tree is responsible for displacing consecutive volumes from the cranium. This pressure wave transmission is expressed by the AVD and is related to the total cranial mobile compliance [16].

A previous study [12] reported that young and elderly healthy adults have similar AVDs and thus suggested that normal ageing is not associated with a change in total intracranial compliance. In this previous work, PC-MRI revealed that normal ageing is associated with a significant reduction of total arterial blood flow and of the systolic peak sharpness, with a related reduction in venous flow. Interestingly, in the same study [12], both cervical and aqueductal CSF SVs were reduced in the elderly subjects, with a constant ratio, suggesting a proportional loss of compliance in these two compartments.

In the current study, patients with neurodegenerative diseases showed a significantly lower AVD (and thus total intracranial compliance) than HEVs. There were no significant differences between the three etiologies. These findings suggest that a-MCI, AD and NPH are probably associated with an imbalance in the dynamic interaction between vascular and CSF compartments. In a-MCI and AD, this imbalance seems to be provoked by abnormal hyperdynamic arterial input, which is dampened to some extent by the venous and CSF regulatory systems. However, our calculations in a-MCI and AD patients did not take account of the brain parenchyma compliance, which may be more impaired here than in primary dynamic disorders like NPH. In contrast, NPH is characterized by primary defects in the regulatory compartments (the intracranial CSF) and, unsurprisingly, the loss of intracranial compliance is evidenced by a change in the AVD.

### The arterial theory of pre-demential MCI and AD states

In patients with clinically defined a-MCI, arterial mean flows were significantly higher than in HEVs. The a-MCI patients also showed a much higher API (measuring the slope of the rise in arterial flow at the beginning of systole), which was consistent with the calculated arterial pulse volume (i.e. the arterial blood volume displaced in early systole). These changes in vascular dynamics may be correlated with the data on the prevalence of hypertension or other cardiovascular risk factors collected on inclusion. We observed mild diabetes in one a-MCI patient and similar proportions of arterial hypertension in the four study groups. Furthermore, all patients and healthy volunteers were only included in the study if their hypertension was medically controlled. Additionally, the a-MCI patients had a lower average heart rate than HEVs, although the difference was not statistically significant, contrasting with the higher arterial blood flow in a-MCI patients (Table 1). These results argue in favour of a change in the regulation of the arterial input (pumped by the heart) in a-MCI patients. An alternative explanation would relate to white matter vascular alterations. Although this parameter was not strictly quantified in the present study, no differences were readily apparent in a visual analysis.

In the absence of confounding parameters, our results suggest that a-MCI predemential states (even those without prominent leukoaraiosis) display a significantly elevated arterial cerebral inflow. These results are concordant with previous studies in which AD patients showed a high prevalence of risk factors for atherosclerosis, supporting the hypothesis that AD is "a vascular disease" [38]. Anatomopathological studies have shown Aβ deposition in the leptomeningeal arteries in AD patients [39] and capillary wall disruption is consistently found [40]. Thus, it seems that increased artery pulsation results in regular dilation and then recoil, driving the interstitial fluid and proteins in the opposite direction to blood flow. The subsequent accumulation of Aβ in the brain's perivascular interstitial fluid drainage pathways could theoretically be responsible for the observed cortical deposits and clinical signs of AD.

The dynamic consequences of this theory have already been investigated in patients with AD. Two studies [41,42] used transcranial Doppler ultrasound to evidence increased intracranial arterial pulsatility in AD patients (1.07 and 0.92), when compared with published reference data (a value of 0.83) [41]. Thus, it seems that the high extracranial arterial pulsatility demonstrated in our study in patients with neurodegenerative disease is transmitted (at least part) to the intracranial vascular compartment (where it can be detected in the basal arteries by using Doppler ultrasound). Elevated extracranial arterial pulsatility has also been suggested by MRI studies; Uftring [21] used mathematical transfer functions to compare the mechanical coupling of vascular and CSF flow oscillations in normal elderly subjects and AD patients. In both groups, there was a tendency for elevated vascular pulsations to cause elevated spinal cord oscillations and reduced subarachnoid CSF oscillations. The amplitude of these changes was greater in AD patients than in normal elderly subjects. Even though Barkhof [33] did not find any changes in vascular or CSF flows in the elderly, we have previously shown [12] that normal ageing is associated with a significant fall in cerebral blood flow and in the pulsatility of the arterial input, as evidenced by a smoother systolic inflow peak than in young adults.

Therefore, our results suggest that a-MCI is associated with an abnormally high arterial pulsatility (independently of white matter abnormalities), which is probably due to degenerative changes in the arterial walls. These hyperdynamic arterial oscillations result in increased pulsatility at the aqueductal level. Indeed, the ventricular CSF SV was higher in a-MCI patients than in HEVs (although the difference did not achieve statistical significance); this is probably a reaction to arterial pulsatility and an attempt to dampen the increase in intracranial pressure.

Our results also revealed greater arterial pulsatility in AD patients, although the difference versus the other groups was not statistically significant. This change may be due to progression of arterial hyperdynamism during the transition from a-MCI to AD and a corresponding, steady reduction of cerebral compliance until a plateau is reached. The elevated arterial pulsatility is accompanied by relevant cerebral atrophy and a proportional decrease in energy needs and thus arterial perfusion. However, the establishment of this hypothesis is hampered by the small size of our groups and needs to be confirmed in larger populations.

### Differential diagnosis for NPH

The vascular results enabled us to discriminate between a-MCI and NPH patients, since the latter had significantly lower arterial flows and pulsatility indexes. In contrast, previously published data [43] have not found arterial pulsation or the aqueductal SV to be of value in differentiating between AD and NPH patients. This discrepancy may be related to differences in selection criteria (i.e. pre-demential a-MCI in our study and patients with advanced AD in Bateman's study). Nevertheless, our results indicate that NPH and AD-like neurodegenerative diseases are probably caused by different etiological mechanisms. As mentioned above, we believe that arterial input may be the prime factor in the dynamic modifications seen in a-MCI; this would result in hyperdynamic functioning of the CSF and venous compartments and a moderate increase in ventricular CSF oscillation. In NPH patients, our results show low arterial pulsation and greatly elevated ventricular CSF oscillation.

In normal subjects, increased intracerebral volume usually caused by systolic arterial inflow, results in expansion of the arterial tree in two directions: (i) outward from the ventricles (with associated outward brain compression and CSF flush flow through the SASs) and (ii) inwards due to ventricle compression and aqueductal CSF flush flow. The elevated arterial inflow in a-MCI patients means that the arteries would essentially expand outward from the ventricles; this would result in greater expansion of the outer portion of the brain and subarachnoid CSF dampening of the pulse pressure. The inward compression of ventricles is moderate and so the aqueductal SV is not greatly elevated.

In NPH, the primary defect has been linked to a decrease in CSF oscillation [27] (and thus compliance) in the cerebral SAS [20] as a result of the compression of cortical venous drainage. Indeed, increased pressures in the cranial sinuses have been demonstrated in animal studies [44]. Reduced CSF absorption in the venous dural sinuses then results in an inward compression of the ventricles and a greater aqueductal SV. The arterial flow reduction seems to be a secondary feature. If this disturbance were to persist, the ventricular system compliance would be overloaded and ventricle dilation would occur.

### The role of aqueductal CSF flow

When comparing the four groups, NPH patients had a significant greater aqueductal SV than the HEVs and AD patients. Previous studies have suggested the usefulness of aqueductal oscillations as a helpful tool for diagnosis of NPH or for selection of patients who will benefit from shunting [27,28]. However, another study reported that aqueductal SV in AD falls between the values recorded in normal subjects and NPH patients and thus cannot be used to differentiate between the two groups of patients [43]. These contradictory results may be due to differences in the respective study populations. Our AD patients were similar in terms of age and cognitive functions to those in the aforementioned study. Although cortical atrophy was not strictly calculated in our study, it appeared to be mild (and thus similar to the volume loss recorded in Bateman's study). The major difference concerns the aqueductal SV values in NPH patients, which differed significantly between the two studies (70 ± 50 in Bateman's study vs. 167 ± 89 in the present study). Our results are similar to those published by Luetmer *et al*. [35] in what was probably a highly selected population. This may be due to different inclusion criteria; gait disorders were mandatory in NPH patients in our study, as this symptom is present in almost 80% of sufferers and is the first of the triad sign to appear. All patients were demented in Bateman's study and gait disorders were unnecessary if incontinence was present.

The difference in aqueductal SV between AD and NPH patients is probably due to different etiological factors. AD is associated with amyloid deposits, which can affect the small cerebral arteries and produce amyloid angiopathy [40]. When combined with the greater prevalence of cardiovascular risk factors in AD patients [15,38], amyloid angiopathy can increase the intrinsic stiffness of the cerebral artery walls and cause failure of the arterial pulse pressure dampening system. In AD, a primary arterial wall pathology may be responsible for greater outward brain expansion, compression of the subarachnoid spaces and thus an increase in spinal cord oscillations [21]. In contrast, it is thought (since publication of the "water hammer effect" theory by Greitz [34]) that the primary etiology in NPH first affects the subarachnoid space and then the arteries, with a reduction in pulse pressure dampening.

## Conclusion

This study has provided a novel vision of cerebral neurodegenerative disease. From a dynamic point of view, the brain constantly interacts with the arterial blood inflow on one hand and venous blood outflow and ventricular and subarachnoid CSF outflow on the other. PC-MRI is a novel way of performing a rapid, non-invasive, reproducible evaluation of these vascular and CSF flows and studying their dynamic coupling throughout the cardiac cycle. This imaging technique can be used to assist with differential diagnoses in neurodegenerative disease. Our results suggest that certain neurodegenerative diseases (i.e. pre-demential a-MCI and AD) may be initiated by an arterial inflow increase, which is the prime factor underlying hydrodynamic deregulation in these patients. In contrast, dramatic, hyperdynamic disturbances of CSF flows were primarily observed in NPH patients.

## Competing interests

The authors declare that they have no competing interests.

## Authors' contributions

SE performed clinical evaluation and selection of patients, image processing and statistical analysis, participated in the study design and drafted the manuscript. OB conceived the study, participated in its design, coordinated the study between hospital departments, performed image acquisition and processing and helped to draft the manuscript. CG performed image acquisition and participated in the data interpretation. AF performed clinical evaluation and participated in patient selection and data analysis. OG, JMS, HD and MEM participated in the design and organization of the study. All authors have read and approved the final version of the manuscript.

## Supplementary Material

Additional file 1 3-**D global animation of CSF and cerebral vascular flows**. This video of an NPH patient was created by merging PC-MRI data sets with morphological MR images. It shows animated CSF/blood flows through the aqueduct of Sylvius, the C2-C3 subarachnoid spaces, the internal and vertebral carotid arteries and the jugular veins during the cardiac cycle. Click here for file
